# Clinical presentation and therapeutic outcome of patients with jackhammer esophagus—a multicenter cohort study in Japan

**DOI:** 10.1007/s10388-022-00916-7

**Published:** 2022-03-06

**Authors:** Hiroko Hosaka, Noriyuki Kawami, Noriaki Manabe, Shiko Kuribayashi, Hiroki Sato, Yasushi Funaki, Maki Ayaki, Ken Hara, Chise Ueda, Tomoaki Matsumura, Yasuhiro Fujiwara, Masafumi Wada, Maiko Kishino, Fumiaki Yano, Tatsuhiro Masaoka, Norihisa Ishimura, Junichi Akiyama, Yorinari Ochiai, Toshio Uraoka, Katsuhiko Iwakiri

**Affiliations:** 1 Department of Gastroenterology and Hepatology, Gunma University Graduate School of Medicine, 3-39-15 Showa-machi Maebashi, Gunma, 371-8511 Japan; 2 Department of Gastroenterology, Nippon Medical School Graduate School of Medicine, Bunkyo-Ku Tokyo, Japan; 3 Division of Endoscopy and Ultrasonography, Department of Clinical Pathology and Laboratory Medicine, Kawasaki Medical School, Kurashiki, Japan; 4 Division of Gastroenterology and Hepatology, Niigata University Medical and Dental Hospital, Niigata, Japan; 5 Department of Gastroenterology, Aichi Medical University School of Medicine, Nagakute, Japan; 6 Division of Gastroenterology and Hepatology, Department of Internal Medicine, Hyogo College of Medicine, Nishinomiya, Japan; 7 Division of Gastroenterology, Department of Internal Medicine, Kobe University Graduate School of Medicine, Kobe, Japan; 8 Department of Gastroenterology, Graduate School of Medicine, Chiba University, Chiba, Japan; 9 Department of Gastroenterology, Osaka City University Graduate School of Medicine, Osaka, Japan; 10 Department of Medicine and Bioregulatory Science, Graduate School of Medical Sciences, Kyushu University, Fukuoka, Japan; 11 Department of Gastroenterological Endoscopy, Tokyo Women’s Medical University, Shinjuku-ku Tokyo, Japan; 12 Department of Surgery, The Jikei University School of Medicine, Minato-ku Tokyo, Japan; 13 Division of Gastroentrology and Hepatology, Department of Internal Medicine, Keio University School of Medicine, Shinjuku-ku Tokyo, Japan; 14 Second Department of Internal Medicine, Shimane University Faculty of Medicine, Izumo, Japan; 15 Department of Gastroenterology, National Center for Global Health and Medicine, Shinjuku-ku Tokyo, Japan; 16 Department of Gastroenterology, Toranomon Hospital, Minato-ku Tokyo, Japan

**Keywords:** Esophageal motility disorders, Dysphagia, Chest pain, Manometry, Eosinophilic esophagus

## Abstract

**Background:**

Jackhammer esophagus (JE) is a hypercontractile esophageal motility disorder diagnosed using high-resolution manometry (HRM). We sought to determine the clinical presentation and therapeutic data of patients with JE in Japan.

**Methods:**

The study included patients with JE, diagnosed through HRM performed for suspicious esophageal motility disorders. Demographics, esophagogastroduodenoscopy, radiology, and therapy data were collected from patient charts.

**Results:**

Among the 4,412 HRM tests performed, 89 patients (61.6 ± 13.4 years; 64 males, 25 females) were diagnosed with JE (2.0%). Dysphagia was the most frequent symptom (80%), followed by chest pain (40%) and heartburn (25%). Esophagogastroduodenoscopy showed abnormal findings in 32% of patients: corkscrew/rosary beads appearance in 26%, narrowing in 11%. Eosinophilic infiltration (> 15 eosinophils/high power field) was diagnosed in 21%. Esophagography showed abnormal findings in 9% of the patients. For the initial therapy, 47 patients received medical treatment followed by peroral endoscopic myotomy (21 patients) and laparoscopic myotomy (two patients). Thirteen patients did not receive any treatment and 10 of those (77%) reported spontaneous resolution of symptoms. Patients who required invasive treatment experienced severe disability in their quality of life and greater maximal distal contractile integral than those who did not.

**Conclusions:**

HRM showed that the prevalence of JE was very low (2%). Esophagogastroduodenoscopy revealed some characteristic features of JE in patients. Some patients showed improvement of symptoms without invasive treatments. Follow-up with/without medical treatment should be considered before performing invasive treatment in patients whose distal contractile integral is relatively low and the quality of life is not impaired.

## Introduction

Jackhammer esophagus (JE) is a hypercontractile esophageal motility disorder diagnosed using high-resolution manometry (HRM). This disorder is defined by the Chicago Classification as a strong contraction, at least two swallows with a distal contractile integral (DCI) > 8,000 mm Hg.s.cm during HRM using the ManoScan system (ManoScan™, Medtronic, Minneapolis, MN, USA).(HRM). [1–3]. The prevalence of JE is very low, and a standard treatment has not been established thus far [1, 4]. Although the pathogenesis of JE remains unclear, cases of eosinophilic infiltration of the esophageal mucosa [4, 5] and eosinophilic myositis have been reported [6, 7]. However, the frequency and etiological significance of eosinophilic infiltration have not been clarified. The low incidence of this disorder renders the accumulation of cases at a single institution difficult. Thus, we conducted this multicenter study to determine the frequency of JE in Japan. The aim of this study was to determine the clinical presentation, findings using esophagogastroduodenoscopy (EGD), radiology, and manometry, and therapy performed in patients with JE in a multicenter retrospective cohort.

## Methods

### Study design and patients

This was a retrospective observational study conducted by the Jackhammer Esophagus Study Group formed by the Japan Esophageal Society. The study involved the review of consecutive HRM studies performed in 16 hospitals in Japan from January 2008 to June 2017. Usual indications for HRM were the detection of esophageal motility disorder in patients with or without symptoms or preoperative analysis of patients with hiatus hernia or gastroesophageal reflux surgery. Patients diagnosed with JE were included in the study. JE was defined based on a DCI cutoff value ≥ 20% of wet swallow according to the Chicago Classification version 3.0 [3]. The cutoff value of DCI was determined according to the HRM systems used: 8,000 mmHg‧s‧cm with the ManoScan system and InSIGHT system (InSIGHT Ultima®, Diversatek, Milwaukee, WI, USA) [8] and DCI > 10,000 mmHg‧s‧cm with the Starlet system (Starlet®, Star Medical, Inc., Tokyo, Japan) [9, 10]. Patients with a history of esophageal surgery were excluded. This study was approved by the research ethics committee of the Japan Esophageal Society and the ethics committee of each participating institution in the study group.

### Fundamental data obtained from each hospital

The total number of patients who underwent HRM during the study period and that of patients with JE were reported from each hospital to calculate the frequency. In addition, the type of HRM equipment was reported.

### Clinical data of patients with JE

Clinical data, manometric, endoscopic, and radiographic findings, and treatment data were collected. Patient charts were reviewed to collect clinical, examination, and therapeutic data. Clinical variables included age, sex, symptoms, and comorbidities. Quality of life (QOL) was rated as no interference with daily life, mild disability, and severe disability (which rendered patients unable to work).

### Endoscopic and radiographic findings

Endoscopic data were reviewed to detect the findings of erosive or eosinophilic esophagitis, and potential findings of esophageal dysmotility (e.g., corkscrew/rosary beads appearance or narrowing). Among the patients who underwent esophageal mucosal biopsy, the detection of > 15 eosinophils/high power field was indicative of eosinophilic infiltration. Radiographic data (i.e., esophagograms) were analyzed to determine the presence of corkscrew/rosary beads appearance in patients. Esophageal wall thickening was assessed by either endoscopic ultrasound or thoracic computer tomography. A total thickness of the esophagus wall > 5 mm indicated wall thickening [11].

### HRM

HRM studies were performed using three types of solid-state HRM systems, i.e., ManoScan system, Starlet system, and InSIGHT system. The catheter was introduced nasally into the esophagus and positioned to record pressures from the hypopharynx to the stomach. The recording was performed with the patient in a supine position; notably, for patients who had difficulty in swallowing, an incline of ≤ 30 degrees was acceptable. HRM was usually performed with a calibration and baseline period without deglutition. Subsequently, ten water swallows (5 mL) were performed with an interval of ≥ 30 s between swallows.

For the calculation of the DCI, only pressures > 20 mmHg were used by the software of each HRM system, i.e., the ManoView™ ESO Analysis Software (Medtronic, Minneapolis, MN, USA, Starlet analysis software (Starmedical, Tokyo, Japan), and BioView Software (Diversatek, Milwaukee, WI, USA). Normal DCI values varied according to the HRM systems, and the cutoff values were extracted from the references. As stated above, we selected 8000 mmHg‧s‧cm for ManoScan [3] and InSIGHT [8], and 10,000 mmHg‧s‧cm for Starlet [10]. Similarly, normal integrated relaxation pressure (IRP) values differed between systems. Concomitant esophagogastric junction (EGJ) outflow obstruction was diagnosed if the median IRP value was higher than the upper limit of each system (i.e., 15 mmHg with ManoScan, 20 mmHg for InSIGHT [8], and 26 mmHg with Starlet [9]). The Chicago Classification separates elevated IRP as EGJ outflow obstruction from JE. However, in cohort studies conducted to date, elevated IRP has also been considered a subtype of JE (i.e., JE with elevated IRP) [12, 13].

### Treatment and clinical outcomes

Information on the treatment and follow-up was also collected. The treatment selection was based on the preference of each treating physician. The treatment efficacy was evaluated by reviewing the patients’ charts using a four-point scale ranging from 1 (complete response) to 4 (no response). Complete response was defined as total disappearance of symptoms. Sufficient/insufficient efficacy was defined as persistence of symptoms, but with sufficient/insufficient improvement. No efficacy was defined as lack of improvement of symptoms after treatment.

### Data and statistical analysis

Statistical analyses were performed using the SPSS version 25.0 (IBM Corp., Armonk, NY, USA) software. Frequency data (sex and symptoms) are expressed as percentages. Continuous data with normal distribution (age and HRM parameters) are expressed as the mean ± standard deviation. Non-normally distributed data are shown as the median with lower and upper quartiles. Student’s *t* test was used for the comparison of parametric data. Continuous parameters were analyzed using analysis of variance, and the Kruskal–Wallis test was utilized as a non-parametric test. Categorical variables were analyzed using Fisher’s exact test. The statistical significance level for all analyses was set at *p* ≤ 0.05.

## Results

### Esophageal manometric findings obtained using each of the three solid-state HRM systems

Based on the 4412 HRM tests performed, 89 patients were diagnosed with JE (2.0%). HRM findings are shown in Table 1. Of the 89 patients with JE, 29, 40, and 20 were diagnosed by ManoScan, Starlet, and InSIGHT, respectively. Mean DCI values for these groups were 8,120.9 mmHg‧s‧cm, 12,041.6 mmHg‧s‧cm, and 7267 mmHg‧s‧cm, respectively. Twenty-one patients (24%) had high IRP over the upper normal limit. Although direct comparison of absolute values was not possible, because each device yields different normal values, there were no significant differences between the devices in the rate of elevated IRP or number of swallows with DCI higher than the normal limit.

**Table 1:** HRM parameters determined using the three different instruments

HRM parameters	ManoScan	Starlet	InSIGHT
Number of patients	29	40	20
EGJ basal pressure	27.7 (18.8, 48.8)	23.6 (15.4, 53.3)	22.9 (17.8, 32.3)
Median IRP	11.1 (7.4, 18.3)	11.85 (7.7, 23.5)	18.0 (13.0, 19.75
Elevated IRP	9 (31%)	8 (20%)	4 (20%)
Mean DCI	8,120.0 (6,822.0, 13,013.0)	12,041.6 (10,473.8, 15,516.75)	7,267.0 (6,005.0, 9,874.3)
Maximal DCI	13,485 (10,168.2, 16,810.0)	18,786.4 (15,787.4, 30,224.5)	15,732.0 (10,673.8, 26,861.3)
Swallows with DCI higher than the limit	5.0 (3.0, 9.0)	3.0 (2.0, 5.75)	5.0 (3.0, 8.0)

Data are presented as the median and interquartile ranges, unless otherwise indicated

*DCI* distal contractile integral, *EGJ* esophagogastric junction, *HRM* high-resolution manometry, *IRP* integrated relaxation pressure

### Patient characteristics

Patient characteristics are shown in Table 2. The mean age was 61.6 ± 13.4 years; 64 males (72%) and 25 females (28%). Dysphagia was the most frequent symptom (80%), followed by chest pain (40%) and heartburn (25%).

**Table 2:** Demographics and clinical characteristics of all 89 patients

Total number of patients	89	
Mean age (years)	61.6 ± 13.4	
Sex (male:female)	64: 25	
Comorbidities		
Neurological disorders	4	(4)
Diabetes	6	(7)
Allergic disorders	25	(28)
Symptoms		
Dysphagia	71	(80)
Chest pain	36	(40)
Heartburn	22	(25)

Data are presented as the number (%) of cases or the mean ± standard deviation

### Endoscopic and radiographic features of JE

EGD was performed in 87 patients (98%); abnormal findings were detected in 32% of the patients: corkscrew/rosary beads appearance in 26% and narrowing in 11% (Table 3). Eosinophilic esophagitis-like findings were observed in seven patients (8%), linear furrows in three patients, rings in one patient, and white exudates in five patients. Biopsies were performed in 58 patients (67%), and esophageal eosinophilia (> 15 eosinophils/high power field) was diagnosed in 12 patients (21%).

**Table 3:** Endoscopic and radiographic features of patients with JE

Endoscopy	*n* = 87	%
Corkscrew/rosary beads appearance	23	26
Narrowing	10	11
EoE-like	7	8
Reflux esophagitis	3	3
Diverticula	2	2
Pseudodiverticula	2	2
Esophageal eosinophilia	n = 58	
> 15 eosinophils/HPF	12	21
0–14 eosinophils/HPF	46	79
Balium esophagogram	n = 71	
Corkscrew	11	15
Rosary beaded	14	20
Narrowing	15	21
Esophageal wall check (CT/EUS)	*n* = 73	
Wall thickening	40	55

Data are presented as the number and % of cases

*CT* computed tomography, *EoE-like* Eosinophilic esophagitis-like, *EUS* endoscopic ultrasound, *HPF* high power field, *JE* Jackhammer esophagus

Esophagography was performed in 71 patients (80%), and abnormal findings were found in 35 of those patients (49%): corkscrew/rosary beads appearance in 25 patients (34%) and narrowing in 15 patients (21%). Esophageal wall thickening, examined by endoscopic ultrasound or chest computed tomography, was observed in 40 patients (55%).

### Treatment and clinical course

Initial treatment is shown in Table 4. Six patients were lost to follow-up and were excluded from the analysis. Forty-seven patients received medical treatment as initial therapy, and sufficient efficacy was recorded in 30 of those (64%). The drugs used in these patients ranged considerably from proton pump inhibitors to antiallergic agents (Table 5). Twenty-one patients (25%) underwent per-oral endoscopic myotomy (POEM), and sufficient efficacy was recorded in 16 of those (76%). Two patients (2%) underwent laparoscopic myotomy; 13 patients (16%) did not receive any treatment and 10 of those (77%) experienced spontaneous improvement of their symptoms.

**Table 4:** Initial treatment and rates of sufficient efficacy (excluding six patients with incomplete follow-up data)

Medicine	Number of patients	Sufficient efficacy*	
	47	30	(64%)
Follow-up without any treatment	13	10	(77%)
POEM	21	16	(76%)
Laparoscopic myotomy	2	2	(100%)

Data are presented as the number (%) of cases

^*^Sufficient efficacy denotes complete response and improvement with sufficient efficacy

*POEM* per-oral endoscopic myotomy

**Table 5:** Types of medical drugs and the rates of patients with sufficient efficacy

	Number of patients (%)		Patients with sufficient efficacy* (%)
PPI/P-CAB	13	(27)	9 (69)
Steroid (systemic)	7	(15)	4 (57)
Steroid (topical)	5	(10)	3 (60)
Calcium channel blockers	18	(38)	11 (61.1)
Nitrates	5	(10)	2 (40)
Antihistamine	5	(10)	5 (100)
Leukotriene receptor antagonist	3	(6)	3 (100)
Potassium channel opener	1	(2)	0 (0)
Shakuyakukanzo-to	13	(27)	9 (69)

Data are presented as the number (%) of cases

Note: Some patients may have received different combinations of drugs as medical treatment

^*^Sufficient efficacy denotes complete response and improvement with sufficient efficacy

*P-CAB* potassium-competitive acid blocker; *PPI* proton pump inhibitor

### Sub-analysis of the group that required invasive treatment

Patients who required invasive treatment, namely POEM or laparoscopic myotomy, were compared with others (Table 6). The rate of requirement of invasive treatment was significantly higher in patients with severe disability in QOL who had difficulty in continuing to work versus others (37% versus 15%, respectively, p 0.026). Comparison of HRM parameters obtained from each device showed that the maximal DCI was significantly higher in patients who required invasive treatment and underwent HRM with the Starlet and InSIGHT systems.

**Table 6:** Comparison between patients who required POEM or laparoscopic myotomy and those who received only medical treatment

	POEM/laparoscopic myotomy (*n* = 27)	Medical treatment/follow-up without therapy (*n* = 62)	
Age, years	61.7 ± 12.5	61.6 ± 13.8	
Sex (male:female)	23:4	41:21	ns
Allergic disease	5 (19%)	20 (32%)	ns
*Symptoms*			
Dysphagia	24 (89%)	47 (76%)	ns
Chest pain	12 (44%)	24 (39%)	ns
Heartburn	7 (26%)	15 (40%)	ns
QOL severe disability	10/27 (37%)	8/55 (15%)	0.026
Wall thickening +	14/25 (56%)	26/47 (55%)	ns
Eosinophil infiltration	2/22 (9%)	10/36 (28%)	ns
*HRM*			
Elevated IRP	5 (19%)	16 (26%)	ns
Swallows with DCI higher than the limit	5 (2–8)	5 (2–8)	ns
*EGJ basal pressure*			
ManoScan	22.95 (15.6, 31)	31.7 (20.15, 48.9)	ns
Starlet	23.2 (16, 37.25)	23.7 (15.7, 53.5)	ns
InSIGHT	19.8 (14.375, 25.75)	27 (18.7, 40.3	ns
*Median IRP*			
ManoScan	12.2 (7.45, 19.1)	10.9 (7.8, 16.9)	ns
Starlet	11.9 (10, 23.8)	11.7 (7.4, 22.1)	ns
InSIGHT	14 (10.75, 16.5)	18 (16.24, 20.75)	ns
*Mean DCI*			
ManoScan	9,705.7 (8,521.0, 14,322.7)	8,029 (6,762.8, 12,309)	ns
Starlet	9,967.2 (8,762, 13,612.5)	9,938 (8,664, 12,678)	ns
InSIGHT	8,519 (6,277.75, 10,290)	7,190.5 (6,036.5, 8,532.5)	ns
*Maximal DCI*			
ManoScan	12,664 (11,367, 20,688.5)	13,556 (10,020.9, 16,289)	ns
Starlet	25,596 (16,841.7, 34,217)	15,583 (13,476, 20,447.1)	0.007
InSIGHT	27,867 (18,541.3, 36,172)	14,327.5 (10,010, 18,596.3)	0.033

Data are presented as the median and interquartile ranges or the mean ± standard deviation

*DCI* distal contractile integral, *EGJ* esophagogastric junction, *RM* high-resolution manometry, *IRP* integrated relaxation pressure, *ns* not significant, *POEM* per-oral endoscopic myotomy, *QOL* quality of life

## Discussion

This was the first multicenter cohort study on JE conducted in Asia. We found that the prevalence of JE in patients undergoing HRM examination in Japan was 2.0%. This is similar to the prevalence (1.7–4.0%) reported in cohort studies from western countries [12–14]. Furthermore, it is almost identical to that reported in a recent meta-analysis [15].

Regarding sex dominance, 72% of the patients analyzed in the present study were males. Numerous previous studies showed that JE was more frequent among females; however, the largest study on JE conducted thus far (227 patients) included mostly males [12]. Hence, our results are not surprising.

The present analysis included endoscopic data for almost all cases. Thirty patients (38%) had findings suggestive of some form of abnormal tonic contraction. The findings of dysmotility observed on barium were also observed on endoscopy. HRM is the gold standard diagnostic test for esophageal motility disorder. However, HRM is still only performed in specialized facilities. Patients with dysphagia or non-cardiac chest pain typically visit general hospitals and undergo EGD. One-third of patients with JE had an abnormal contraction which could be detected by EGD; this would be a good reason for referral to HRM examination. However, since two-thirds of the patients did not have abnormalities detected through endoscopy, it is recommended that patients with long-lasting symptoms undergo HRM.

A notable finding of this study was that a high percentage of patients with JE had eosinophilic infiltration in esophageal mucosa. It has been reported that eosinophilic esophagitis causes a variety of esophageal motility disorders, ranging from aperistalsis to strong contractions and achalasia-like dysmotility [16, 17]. Roman et al. reported eosinophilic infiltration into the esophageal mucosa in three of 41 patients (7%) with JE [1]. Philonenko et al. reported eosinophilic infiltration into the esophageal mucosa in three of 63 (4.7%) patients with JE who underwent esophageal biopsy [12]. Furthermore, there is an increasing number of case reports of JE accompanied by eosinophilic esophagitis [5, 18] and eosinophilic myositis [6, 7]. In many of these cases, eosinophilic myositis is detected by muscle layer biopsy during POEM [6, 7, 19]. Furthermore, from the therapeutic perspective, treatment with steroids has been successfully utilized in these patients [18, 20]. In the present study, we found eosinophilic infiltration (i.e., > 15 eosinophils/high power field) in 12 of the 58 patients (21%) with JE who underwent esophageal mucosal biopsies. Since the frequency of eosinophilic esophagitis among those undergoing examination with EGD in Japan is < 1% [21, 22] eosinophil infiltration in patients with JE appears to be more common. Of the 12 cases with proven eosinophil infiltration, 9 were treated with steroid therapy (5 systemic and 4 local), and 5 of those (56%) had satisfactory improvement of symptoms.

Although a standard treatment for JE has not been established, POEM is increasingly being used. According to a meta-analysis, 82% of patients treated with POEM showed improvement in symptoms [15]. In this study, 76% of patients who underwent POEM as initial treatment reported satisfactory symptom improvement. Nevertheless, 77% of patients who did not receive treatment also experienced improvement in symptoms during the follow-up. In terms of QOL among these 13 patients, 12 patients had mild disability or no interference and one patient experienced severe disability.

The selection of treatment for patients with JE appears to be a challenging task, as some of them in the present study improved without receiving medical treatment. Based on the characteristics of patients who required invasive treatment, POEM and myotomy were necessary in those with severe disability in terms of QOL as well as higher maximal DCI, who underwent HRM examination with the Starlet and InSIGHT systems. It has been reported that high DCI is an indicator for the requirement of POEM in JE [23]. Notably, there was no difference in the highest DCI among patients examined with the ManoScan system. Although the reason for this observation is unclear, it may be attributed to the low number of cases which required invasive treatment among those examined with this system, as well as inconsistent treatment selection across hospitals. Only six (21%) of those tested with the ManoScan system received invasive treatment versus 15 (38%) and six (30%) of those tested with the Starrett system and InSIGHT system, respectively. The present data showed that patients with mild symptoms and mild elevation in DCI may not require invasive therapy during the follow-up period. Therefore, our results indicate that POEM is not applicable to all cases of JE; medical observation, including follow-up, is an option for patients with mild impairment of QOL.

This study revealed that the types of medical treatment administered varied. Steroids and antiallergic drugs can be used to treat eosinophilic involvement, while other agents can be utilized to reduce smooth muscle contractility. Calcium blockers were the most commonly used pharmacological agents, and their efficacy was good in approximately 60% of patients in this study. It has been proposed that the pathogenesis of JE is based on an imbalance between inhibitory and excitatory innervation. Some studies reported that JE may develop due to possible injury to the vagus nerve during lung transplantation and radiofrequency catheter ablation for atrial fibrillation [24, 25]. A physiological study also demonstrated that cholinergic nerve excitation is associated with strong contractions, providing evidence that anticholinergic agents may be effective in the treatment of JE [26].

Shakuyakukanzo-to, a Japanese herbal medicine, is widely used for the treatment of muscle cramps in Japan. Some case reports have also shown its effectiveness in JE [23, 27]. It has been demonstrated that shakuyakukanzo-to exerts an antispasmodic effect on smooth muscle and inhibits peristalsis of the digestive tract in animals [28]. Moreover, in humans, it inhibits gastrointestinal motility when sprayed directly into the intestinal tract during endoscopy [29]. Therefore, this agent may be a new treatment option for JE.

This study had several limitations. Firstly, there was no uniform treatment strategy, and the selection of treatment depended on each treating physician. Since this was a retrospective study, it was difficult to objectively evaluate subjective data (e.g., symptoms and treatment efficacy). Moreover, we could not evaluate the effect of each drug, because several drugs were used simultaneously in combination. Prospective studies are warranted to determine the most appropriate treatment strategies. Second, this study examined three different HRM devices. We could not compare the parameters of HRM obtained for all patients together, as the values of the measured parameters depend on the type of device used.

In conclusion, the present data demonstrate that the prevalence of JE in Japan is almost identical to that reported in western countries. Patients showed some characteristic features of JE during examination with EGD. Many patients showed improvement in their symptoms without undergoing invasive treatments, such as POEM. We suggest that follow-up with or without the administration of medical treatment should be considered prior to performing invasive treatment in patients with a relatively low DCI and not severe impairment in QOL. Further prospective study is warranted to determine the most appropriate treatment strategy.
